# Communicative Social Intentions Modulate Emotional Mimicry Responses

**DOI:** 10.1111/psyp.70137

**Published:** 2025-09-09

**Authors:** Leon O. H. Kroczek, Silke Frank, Uta Gold, Fridolin Hesse, Selina Hettenkofer, Nadja Peterreins, Lorenz Teutsch, Valerie Theophile, Andreas Mühlberger

**Affiliations:** ^1^ Department of Psychology, Clinical Psychology and Psychotherapy University of Regensburg Regensburg Germany

**Keywords:** digital communication, EMG, emoticons, emotion, psychophysiology, social interaction, virtual agents

## Abstract

Facial emotional expressions are interactive signals that communicate intentions. Previous research has shown that sending a facial emotional expression influences the evaluation of response expressions, but the mechanisms behind this effect remain unclear. In a preregistered experiment, 68 participants were asked to send an emoji (happy, neutral, and angry) to a virtual agent in front of them, whereupon the agent reacted with either a smiling or frowning facial expression. Valence and arousal ratings were obtained and mimicry responses to the agent's expression were measured via facial EMG of the Zygomaticus and Corrugator muscles. The results show that being smiled at is more pleasant and elicits greater Zygomaticus activation when the smile is received as a response to a happy emoji compared to an angry emoji. In contrast, being frowned at is less pleasant and increases Corrugator activity when the angry expression is received as a response to a happy emoji compared to an angry emoji. Finally, we found that sending an emoji resulted in activation of facial muscles corresponding to the valence of the emoji. The results support the role of affiliative mechanisms in the exchange of facial expressions but also demonstrate that persons are sensitive to the congruency between emotional signals of sender and receiver. These effects might be driven by physiological feedback. By implementing digital emotional expressions, the present study dissects the communicative act from the motor display of a facial expression and thus allows us to probe mechanisms behind social interactions in real and digital worlds.

## Impact

Communicating emotional intentions via emojis influences how people evaluate and mimic facial expressions during social interactions. Sending a smile emoji increased pleasantness and mimicry of happy facial responses, while sending an angry emoji had the opposite effect. Importantly, sending emojis activated corresponding facial muscles, suggesting a potential physiological mechanism underlying digital emotional communication. These findings advance our understanding of how communicative intentions shape social–emotional processing in both face‐to‐face and digital interactions.

## 1 Introduction

Facial emotional expressions are important for face‐to‐face social interactions. While facial emotional expressions can reflect a person's affective response to a stimulus, it has been argued that in interpersonal encounters, communication is the prime function of facial emotional expressions (Jack and Schyns 2015). Here, expressing emotions allows one to communicate intentions and affective states to an interaction partner. According to the emotion as social information model (EASI), emotional expressions can inform social processes via two pathways (Van Kleef 2009). On the one hand, the observation of an emotional expression can elicit an affective reaction that helps to understand the state of another person. On the other hand, emotional expressions can be used to draw inferences about the state, intention, and characteristics of the person expressing the emotion, but also the situation and the self (Lange et al. 2022; Van Kleef 2009). Based on these inferences, the observer can then predict upcoming behavior and prepare an adaptive response, allowing for efficient and well‐coordinated social interactions (Cheshin et al. 2016; Kroczek et al. 2021, 2024). Importantly, social interactions do not stop at this point, but the perception of another person's emotional expression may also elicit an emotional expression in the original observer, which then again evokes inferential processing in the original sender. This results in a back‐and‐forth of sending and receiving emotional expressions where both interactive partners process nested representations about how current expressions can be inferred from the history of preceding expressions (Lehmann et al. 2023). This emphasizes the interactive nature of social–emotional processing.

Mimicry can be described as a form of interactive behavior where the behavior of one person is imitated by another person (Chartrand and Bargh 1999; Chartrand and Lakin 2013) and emotional mimicry relates to the imitation of emotional expressions, such as facial emotional expressions (Dimberg 1982; Fischer and Hess 2017). Emotional mimicry has been discussed as a communicative signal itself (Sato et al. 2008) and has been related to affiliative functions by increasing closeness and liking in social relations (Hess and Bourgeois 2010; Salazar Kämpf et al. 2018; Wicher et al. 2025). Importantly, emotional mimicry is dependent on the social context (Seibt et al. 2015). It has also been argued that emotional mimicry is driven by emotional intent and is only observed when interactive partners have an affiliative intent or goal (Hess 2021). However, a recent study found that emotional mimicry did not differ between agreeing and disagreeing dyads (Ravreby et al. 2024). This suggests that emotional mimicry could help to increase the understanding of another person's emotions by instating the emotional expression in the observer even without an affiliative intent. In this case, proprioceptive feedback about one's emotional expression could influence emotional processing (Efthimiou et al. 2024; Flack 2006). Overall, emotional mimicry can be seen as a form of interactive and communicative behavior with important social functions.

Even with mimicry being an interactive process, paradigms investigating mimicry and social interaction typically only implement a one‐way direction of social exchange, that is, the impact of the experimental stimuli on the participants, while the other direction, that is, the impact of participants behavior on the experimental environment, is missing. Recent studies have used a minimal interactive paradigm in which persons were asked to send a facial emotional expression to a virtual agent presented on the screen who then responded with another facial emotional expression (Kroczek and Mühlberger 2022, 2025). Results from this paradigm showed that sending an affiliative (smile) compared to a non‐affiliative emotional expression (frown) towards the interactive partner increased the experienced pleasantness and the Zygomaticus activation of a happy response expression. Furthermore, EEG data in a similar paradigm demonstrated enhanced processing of the agent's emotional response expression only after sending an emotional but not neutral facial expression, suggesting more elaborate processing of facial emotions when they become relevant in an interaction exchange (Kroczek and Mühlberger 2025). These data show that communicating emotions modulates socio‐affective processing of facial expressions in interactions and is in line with an affiliative mechanism of reciprocal facial expressions.

The exact mechanism by which sending a facial expression influences the processing of the response expression, however, remains unclear. There are two possible explanations that could underlie such a process. First, the physiological (pre‐)activation of a facial muscle could impact an agent's affective state via proprioceptive feedback mechanisms which might then influence the affective processing of the emotional facial expression received in response (Efthimiou et al. 2024; Flack 2006). Alternatively, sending a facial emotional expression may activate higher‐order representations relating to one's intention (to affiliate) and the pre‐activation of this representation may then modulate processing of the response expression. Such representation‐guided processing has been proposed as a mechanism for action understanding (Caramazza et al. 2014; Tucciarelli et al. 2019). Furthermore, this can also be linked to predictive models of interpersonal behavior which suggest that agents predict another person's behavior based on representations of previous interactive turns (Lehmann et al. 2023). Previous paradigms were not able to differentiate between these explanations as the intention and the physiological pre‐activation were both mediated via facial emotional expressions.

Importantly, facial emotional expressions are not the only means by which intentions can be conveyed. With the rise of digital communication, other forms of communicative cues have been developed to signal intentions in (digital) social interactions. Emojis are a key example in this regard as they allow one to convey emotions and add non‐verbal information to text messages (Cherbonnier and Michinov 2022). Recent findings demonstrate that emojis may be functionally equivalent to facial emotional expressions in enhancing emotional valence and providing social information about the goals and intentions of the person using the emoji (Erle et al. 2022). Altogether, emojis provide experimenters with the opportunity to have participants send emotional signals without the need to actively produce a facial emotional expression.

Building on this research, the present study implemented emojis as effective interpersonal signals to display intentions in (virtual) face‐to‐face social interactions, allowing us to manipulate communicative emotional intentions without having participants explicitly display facial emotional expressions. Crucially, as emotional expressions can be linked to particular social motives (Parkinson 2005), the display of an emotional expression can be thought of as the communicative signal of an intention, even when the true intention of the sender is different. This new approach has several benefits. First, it allows for investigating the effects of sending an emotional expression to another person after dissecting the communicative act from the motor display. This allows for investigating the underlying mechanisms of interactive social emotional exchanges. Second, on a methodological level, sending digital rather than real expressions reduces the strong muscular activation that is elicited when one actually smiles or frowns (Kroczek and Mühlberger 2022). This increases the sensitivity of EMG measurements to responses elicited by the interactive partner; that is, mimicry responses, thus making it more suitable to study psychophysiological effects in interactive paradigms.

Consequently, the goal of the present study was to investigate whether communicating an emotional intention via emoji influences mimicry and social evaluation. An interactive paradigm was implemented in which participants were face‐to‐face with a virtual agent on a screen (Kroczek and Mühlberger 2022). Participants were instructed to send an emotional signal toward the virtual agent. In contrast to Kroczek and Mühlberger (2022), this emotional signal was conveyed by sending an emoji (angry, neutral, happy) rather than displaying a facial emotional expression. Following the emoji, the virtual agent then displayed a facial emotional expression (smile or frown). Activation of the Zygomaticus and Corrugator muscles elicited by the agent's expression was measured as dependent variables. In addition, we assessed participants evaluation of the social interactions via ratings of valence and arousal. In a set of preregistered hypotheses, we expected to conceptually replicate the effects of Kroczek and Mühlberger (2022). First, we hypothesized that sending a happy (smiley emoji) compared to an angry emotional signal (frowny emoji) would increase the valence of interactions in which the agent responded with a happy facial expression but not the valence of interactions in which the agent responded with an angry facial expression. Next, happy facial expressions were expected to be evaluated generally as more pleasant than angry expressions. Furthermore, we expected an interaction between sending an emoji and receiving an emotional facial expression on activation of the Zygomaticus muscle, with an increase in activation when sending a happy emoji compared to sending an angry emoji that was answered with a happy facial expression, but not when answered with an angry facial expression. With respect to the Corrugator muscle, we expected increased activation for angry relative to happy facial expressions, but no modulation with respect to the sent emoji. This differentiation between the Zygomaticus and Corrugator muscles was informed by previous studies which found interaction effects between sending and receiving on emotional mimicry only for the Zygomaticus but not the Corrugator muscle (Hess and Bourgeois 2010; Kroczek and Mühlberger 2022, 2025).

## 2 Methods

### 2.1 Participants

A total of 75 healthy volunteers participated in the study. Seven participants were excluded from data analysis (*n* = 2 due to problem during data acquisition, *n* = 1 because they did not follow experimental instructions, *n* = 4 due to excessive artifacts in the EMG signal). The final sample consisted of 68 participants (55 female, M_age_ = 22.74 years, SD_age_ = 5.71, age range = 18–50). This sample size corresponded to the pre‐determined sample based on a previous study in which the interaction of sending and receiving facial emotional expressions was investigated (Kroczek and Mühlberger 2022) and allows for detecting small‐to‐medium effect sizes (*d* = 0.35) with a power of 0.80. Participants did not report mental or neurological disorders and had normal or corrected‐to‐normal vision. All participants gave written informed consent. The study protocol was in line with the Declaration of Helsinki and was approved by the ethics committee of Regensburg University. Participants received course credit for participation.

### 2.2 Material

Stimuli consisted of video clips with a duration of 6 s displaying one of four different virtual agents (two men and two women) in front of a black background. Virtual agents were created with MakeHuman (v 1.1.1, www.makehuman.org) and the animation sequence was edited and rendered in Blender (v2.79, Blender Foundation, Amsterdam, the Netherlands). A detailed description of stimulus creation is presented in Kroczek and Mühlberger (2022). Video clips showed each virtual agent displaying a happy or an angry facial emotional expression. Additionally, five different versions for each agent and emotional expression were created which varied agents' eye blinks and slight head movements in order to increase the naturalism of the stimuli. This resulted in a total of 40 experimental stimuli. Timing was kept constant in all video clips. In the initial 4000 ms of each video clip, agents displayed a neutral expression. The neutral expression then changed to a happy or angry facial expression within 500 ms and was displayed in full for another 1500 ms.

In order to test how the emotional expressions were experienced in general, a post‐test was conducted in which participants were asked to rate still frames of a happy, angry, and neutral facial expression with respect to valence and arousal for each agent. Analysis of these ratings confirmed that the facial emotional expressions were experienced as intended; that is happy expressions were more pleasant and arousing than neutral expressions, and angry expressions were less pleasant and more arousing than neutral (see Figure S1).

Three emojis (iOS sourced) were selected to signal participants emotional intention. For happy, the emoji “Grinning Face” (U + 1F600); for neutral, the emoji “Neutral Face” (U + 1F610); and for angry, the emoji “Angry Face” (U + 1F620) were selected.

### 2.3 Experimental Design and Procedure

The study implemented a 2 × 3 within‐subject design with the factors *Emoji* (sent‐happy, sent‐neutral, sent‐angry) and *Response Expression* (happy, angry). *Emoji* indicates the emoji that was sent by the participant to the virtual agent, while *Response Expression* relates to the facial emotional expression that was displayed by the virtual agent following the emoji. All six conditions were presented in pseudo‐randomized order with 20 trials per condition, resulting in a total of 120 trials.

At the beginning of the study, participants were instructed that they would interact with virtual agents during the experiment and that they would be asked to direct an emotion towards the agent by sending an emoji, and that the agent would react to that emoji by displaying a facial emotional expression. Participants then completed a demographic questionnaire, the Social Phobia Inventory (Connor et al. 2000), and the Positive and Negative Affect Schedule (PANAS, Watson et al. 1988). Data from the Social Phobia Inventory and the PANAS are part of a bigger research project and are not analyzed in the current study but are available in the online repository (for descriptive data see Table S1). Next, EMG electrodes were attached to the face (see below) and participants were seated in front of a screen (21.5‐in. LCD, HP E221c, 1920 × 1080 resolution, 60 Hz) at a distance of 50 cm.

**FIGURE 1:**
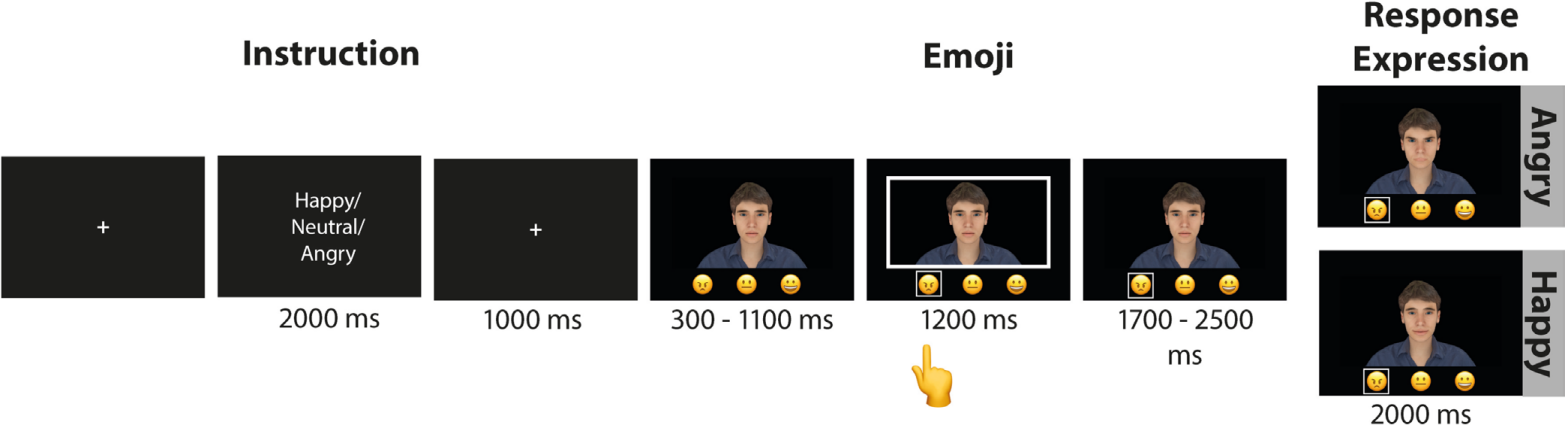
Trial structure. The instruction informed participants about which emoji they had to send to the agent when the cue stimulus was presented. The white rectangular frame around the agent cued participants when to send the emoji via button press. The virtual agent then responded with a happy or angry facial.

The experiment was controlled using Psychtoolbox‐3 (Pelli 1997) in Matlab 8.6 (MathWorks, Natik, MA, USA). Participants underwent five practice trials before the start of the actual experiment. The schematic trial structure is presented in Figure 1. Each trial started with the presentation of a fixation cross for 1000 ms. Participants were then instructed about the emotion that they had to communicate towards the virtual agent. Therefore, an emotion adjective (Happy, Neutral, Angry) was displayed on the screen for 2000 ms. Following another fixation cross of 1000 ms, the video clip was presented on the screen (centered with a size of 1519 × 854 px) displaying a frontal view of the virtual agent. Below the agent, the angry, neutral, and happy emojis were displayed from left to right. After a random interval of 300–1100 ms, a white rectangle was displayed around the agent. The onset of the rectangle cued participants to send the instructed emoji via button press (buttons were marked on the keyboard and corresponded to the left–right position of the emoji on the screen, Y = angry, V = neutral, M = happy, using a German QWERTZ keyboard). The rectangle was visible for a total of 1200 ms, and participants were informed to send the emoji during this interval. After the white rectangle disappeared, the agent remained with a neutral expression for another 1700–2500 ms. Then, exactly 4000 ms after the onset of the video clip, the expression of the virtual agent changed from neutral to an emotional expression (happy or angry) with a transition length of 500 ms and was then displayed for another 1500 ms until the end of the video clip. After the video clip ended, a new trial was presented, or in 20% of all trials, ratings were obtained (catch trials).

There were four catch trials per condition in which ratings of valence (“How pleasant or unpleasant did you feel with the previous person?”) and arousal (“How high was your emotional arousal with the previous person?”) were measured by using a 7‐point Likert scale (from 1 = very low/very unpleasant to 7 = very high/very pleasant). Ratings were entered via mouse click. Rating questions explicitly referred to valence and arousal with respect to the agent in order to measure participants' evaluation of the complete interaction with the virtual agent and not only of the facial expression.

### 2.4 EMG Recording and Preprocessing

EMG activation of M. Zygomaticus major and M. Corrugator supercilii was continuously measured throughout the experiment. For each muscle, two 6 mm Ag/AgCl electrodes were attached to participants left half of the face, and an additional ground electrode was attached to the middle of the forehead following the guidelines of Fridlund and Cacioppo (1986). Skin was prepared with alcohol and an abrasive paste (Skin‐Pure, Nihon Kohden, Tokio, Japan) and impedances were kept below 50 kΩ (mean = 11.08 kΩ, SD = 6.58). The left mastoid served as an online reference during recording. Data was acquired with a sample rate of 1000 Hz using a V‐Amp amplifier (BrainProducts, Gilching, Germany).

Data preprocessing was conducted in Matlab v 8.6 using the Fieldtrip toolbox (v20220714, Oostenveld et al. 2011). Electrodes of each muscle were first referenced to each other, then bandpass filtered between 30 and 500 Hz, and an additional notch filter at 48–52 Hz was applied. Filters were windowed‐sinc finite impulse response filters (−6 dB, half amplitude, hamming window). Data were then rectified and integrated using a moving window average with a length of 125 ms. In the next step, data were segmented into epochs timelocked to the onset of the agents' response expression from 500 ms pre onset to 2000 ms post onset. Additional segments were created to analyze EMG response during the sending of the emoji. Here, epochs were timelocked to the onset of the cue which informed participants to send the emoji. All epochs were baseline corrected with the 500 ms window pre onset serving as the baseline window. Epochs in which participants had not sent the correct (i.e., instructed) emoji were rejected (mean trials = 2.61, SD = 9.90). In addition, epochs were rejected when activation exceeded ±8 mV in the baseline period or ±50 mV in the post onset window (mean trials = 1.43, SD = 2.90). Data were then z‐transformed in order to account for deviation in normality as well as differences between muscles (Bush et al. 1993). Transformation was applied within participant and muscle and across conditions (Rutkowska et al. 2024). Finally, data of each muscle was averaged over trials of the same condition and exported in 500 ms windows for statistical analyses.

### 2.5 Statistical Analyses

Statistical analyses were conducted in the R environment (v4.1.1; R Core Team 2021) using the packages ez (Lawrence 2016) and tidyverse (Wickham et al. 2019). Arousal and valence ratings were analyzed separately using repeated measurements ANOVAs with the factors Emoji (3) and Response Expression (2). Statistical analysis of the EMG data was conducted separately for Zygomaticus and Corrugator muscles in time‐windows of 500 ms length (i.e., [0–500], [500–1000], [1000–1500], [1500–2000]). EMG data was then analyzed using repeated measurement ANOVAs with the factors Emoji (3), Response Expression (2), and Time (4). Violations of sphericity were corrected using the Greenhouse–Geisser method (Greenhouse and Geisser 1959). Significant interactions were investigated by post hoc *t*‐tests using the Holm method to control for multiple comparisons (Holm 1979).

### 2.6 Open Science Statement

Study procedures, hypotheses, analyses were preregistered before data creation (https://osf.io/sqtpe). All data, materials, and analysis scripts are available in a public repository (https://osf.io/z8cve/).

## 3 Results

### 3.1 Manipulation Check: Emotional Meaning of Emojis

**FIGURE 2:**
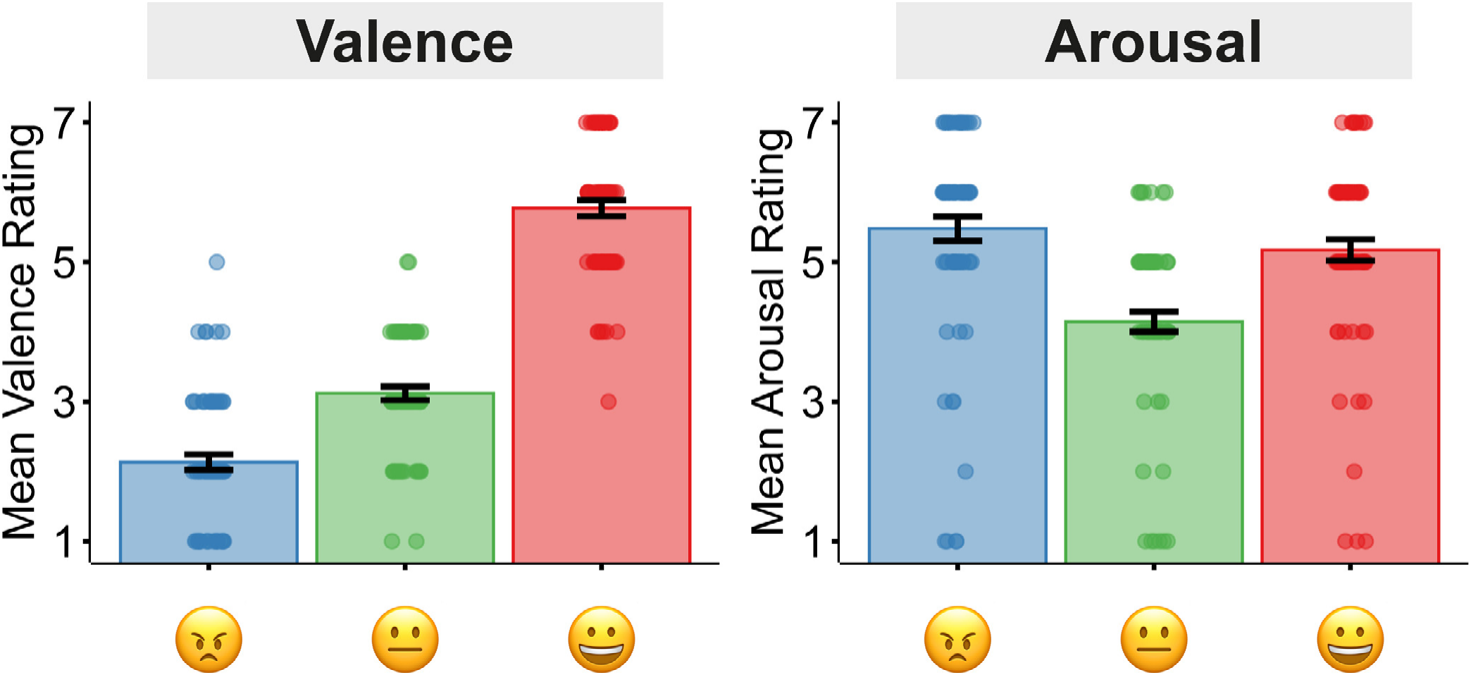
Valence and Arousal ratings of emojis. Ratings were given on a Likert scale from 1 to 7. Error bars reflect the standard error of the mean.

To test whether participants associated emojis with specific emotional meanings, we assessed ratings of valence and arousal for each emoji (Figure 2). Results demonstrate that a happy emoji was more pleasant than a neutral emoji, *t*(67) = 15.92, *p* < 0.001, *d* = 1.93, while an angry emoji was less pleasant than a neutral emoji, *t*(67) = −7.46, *p* < 0.001, *d* = 0.90. With respect to arousal, comparisons revealed that both happy and angry emojis were more arousing than the neutral emoji (happy: *t*(67) = 5.49, *p* < 0.001, *d* = 0.67, angry: *t*(67) = 7.04, *p* < 0.001, *d* = 0.85), but there was no difference between angry and happy emojis, *t*(67) = 1.71, *p* = 0.092, *d* = 0.21. All in all, the emojis were associated with distinct emotional meanings, making them suitable to communicate emotional intent.

### 3.2 Valence

**FIGURE 3:**
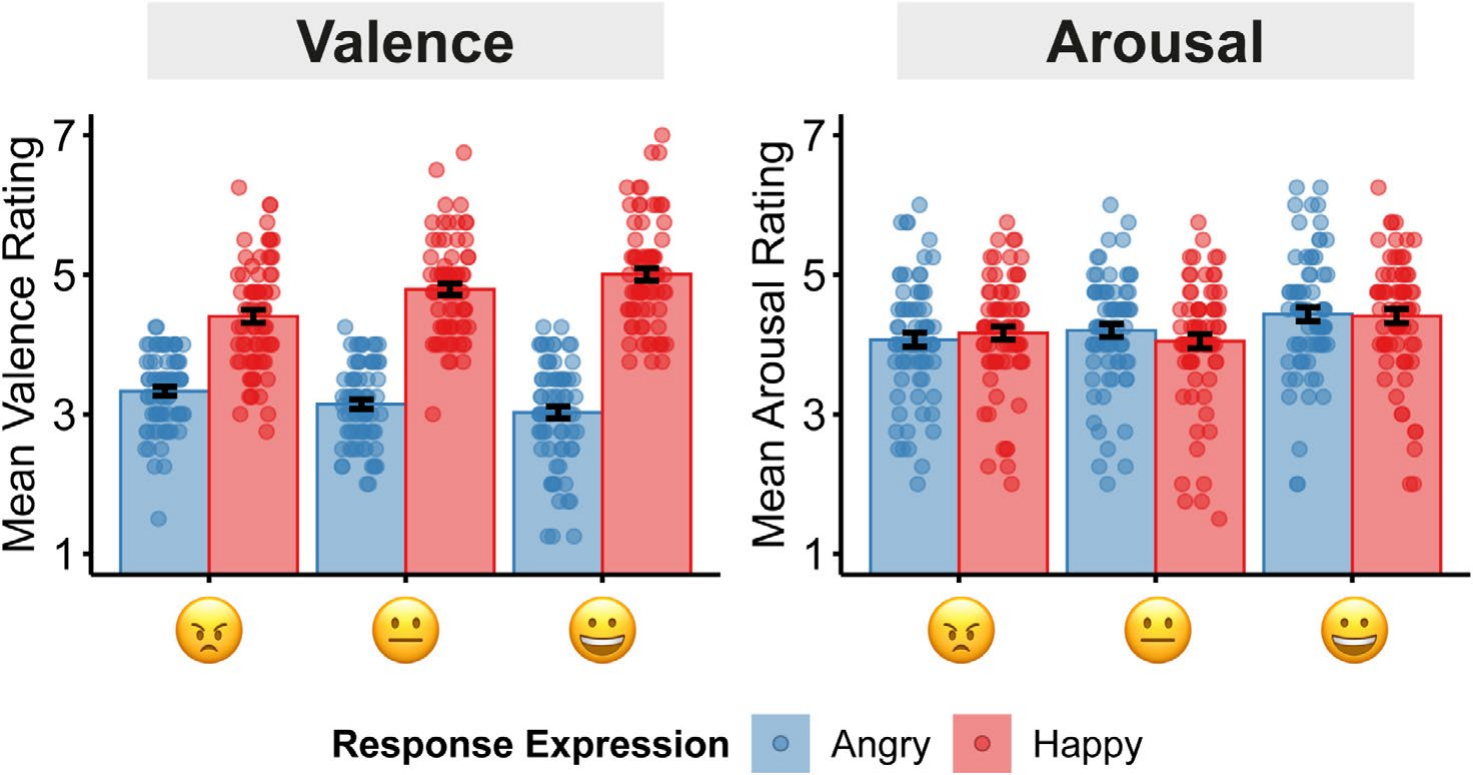
Valence and Arousal ratings. Data are displayed as a function of the emoji that was sent by the participant (*x*‐axis), and the facial emotional response expressions of the virtual agents (blue = angry, red = happy). Ratings were given on a Likert scale from 1 to 7. Error bars reflect the standard error of the mean.

We investigated how the interplay of sending an emoji and receiving a facial emotional expression in response affected participants' experience of valence of the social interaction (Figure 3). A repeated measures ANOVA revealed a main effect of Response Expression, *F*(1, 67) = 144.43, *p* < 0.001, η_p_^2^ = 0.68, with happy response expressions being more pleasant than angry response expressions. While there was no main effect of Emoji, *F*(1.78, 119.29) = 2.70, *p* = 0.078, η_p_^2^ = 0.04, there was a significant interaction effect between Emoji and Response Expression, *F*(1.80,120.37) = 30.94, *p* < 0.001, η_p_^2^ = 0.32. To investigate the interaction effect, we tested the influence of emojis on the evaluation of valence for happy and response expressions separately. For happy response expressions, interactions were rated as most pleasant when participants had sent a happy emoji, as intermediate when participants had sent a neutral emoji, and as least pleasant when participants had sent an angry emoji (sent‐happy vs. sent‐neutral: *t*(67) = 3.12, *p* = 0.005, *d* = 0.38; sent‐happy vs. sent‐angry: *t*(67) = 5.92, *p* < 0.001, *d* = 0.72; sent‐neutral vs. sent‐angry: *t*(67) = 3.95, *p* < 0.001, *d* = 0.48). Interestingly, a reversed pattern was observed for angry response expressions. Here, valence was reduced when a happy emoji had been sent compared to when a neutral, *t*(67) = −3.27, *p* = 0.005, *d* = 0.39, or angry emoji had been sent, *t*(67) = −4.30, *p* < 0.001, *d* = 0.52. There was no difference between a sent‐happy or sent‐neutral emoji, *t*(67) = −1.62, *p* = 0.108, *d* = 0.20. Overall, the results demonstrate that sending an emoji impacts the evaluation of the response expression. Happy expressions were evaluated as more pleasant when in response to a happy compared to a neutral or an angry emoji, while angry expressions were evaluated as more unpleasant when in response to a happy compared to an angry emoji.

### 3.3 Arousal

A repeated measures ANOVA was conducted to investigate the effects of sending an emoji and receiving a facial emotional expression on arousal ratings (Figure 3). The analysis revealed only a main effect of Emoji, *F*(2,134) = 20.22, *p* < 0.001, η_p_^2^ = 0.23, but no main effect of Response Expression, *F*(1,67) = 0.01, *p* = 0.932, η_p_^2^ < 0.01, and no interaction effect, *F*(2,134) = 2.40, *p* = 0.094, η_p_^2^ = 0.03. Post hoc t‐tests revealed that sending a happy emoji was more arousing than sending a neutral emoji, *t*(67) = 5.66, *p* < 0.001, *d* = 0.69, or an angry emoji, *t*(67) = 5.39, *p* < 0.001, *d* = 0.65, but there was no difference between sending an angry and a neutral emoji, *t*(67) = 0.26, *p* = 0.796, *d* = 0.03. Overall, sending a happy emoji increased arousal, but the interplay of sending and receiving emotional expressions did not affect arousal.

### 3.4 M. Zygomaticus

**FIGURE 4:**
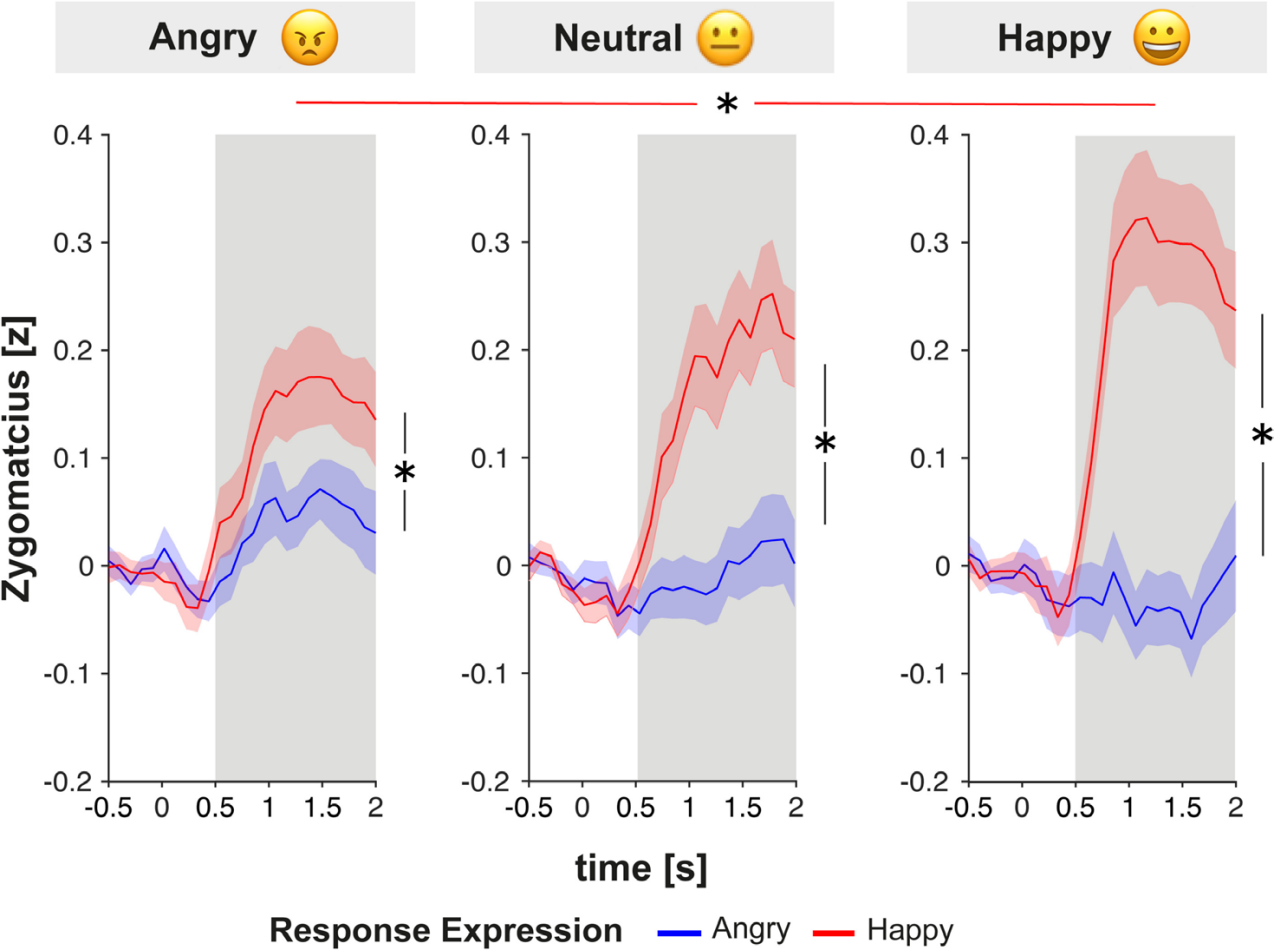
Zygomaticus activation elicited by the facial emotional expression of the agent as a function of the emoji that was sent by the participant (left: Angry, middle: Neutral, right: Happy). Shaded areas reflect the standard error of the mean.

Zygomaticus activation was first analyzed using a repeated measures ANOVA with the factors Emoji, Response Expression, and Time (including 4 levels of non‐overlapping 500 ms windows from 0 to 2000 ms post onset of the response expression). Because the ANOVA revealed a significant three‐way interaction, *F*(4.16,278.41) = 4.14, *p* = 0.002, η_p_^2^ = 0.06, a step‐down analysis was conducted to investigate the interaction of Emoji and Response Expression in each time window (see Tables S2 and S3 for full list of effects). We found significant interaction effects between Emoji and Response Expression in all but the earliest time window (0–500 ms: *F*(2,134) = 0.03, *p* = 0.975, η_p_^2^ < 0.01; 500–1000 ms: *F*(2, 134) = 4.39, *p* = 0.014, η_p_^2^ = 0.06; 1000–1500 ms: *F*(2, 134) = 8.93, *p* < 0.001, η_p_^2^ = 0.12; *F*(2, 134) = 4.52, *p* = 0.013, η_p_^2^ = 0.06). Consequently, post hoc *t*‐tests were conducted for data averaged within 500–2000 ms post onset of the response expression. While Zygomaticus activation was higher for happy compared to angry response expressions in all emoji conditions (all *p* < 0.05), Zygomaticus activation for happy but not angry response expressions differed as a function of the emoji. Specifically, we found higher Zygomaticus activation for happy response expressions when participants had initiated the interaction with a sent‐happy emoji compared to a sent‐angry emoji, *t*(67) = 2.89, *p* = 0.031, *d* = 0.35. There was no significant difference in Zygomaticus activity for happy responses between sent‐happy and sent‐neutral emoji conditions, *t*(67) = 2.10, *p* = 0.158, *d* = 0.25, and between sent‐neutral and sent‐angry emoji conditions, *t*(67) = 1.00, *p* = 0.646, *d* = 0.12. There was also no difference between emoji conditions for angry response expressions (all *p* > 0.05). Overall, sending a happy versus angry emoji specifically resulted in higher Zygomaticus activation for happy response expressions (Figure 4), while sending a neutral emoji was in between the sent‐angry and sent‐happy emoji conditions.

### 3.5 M. Corrugator

**FIGURE 5:**
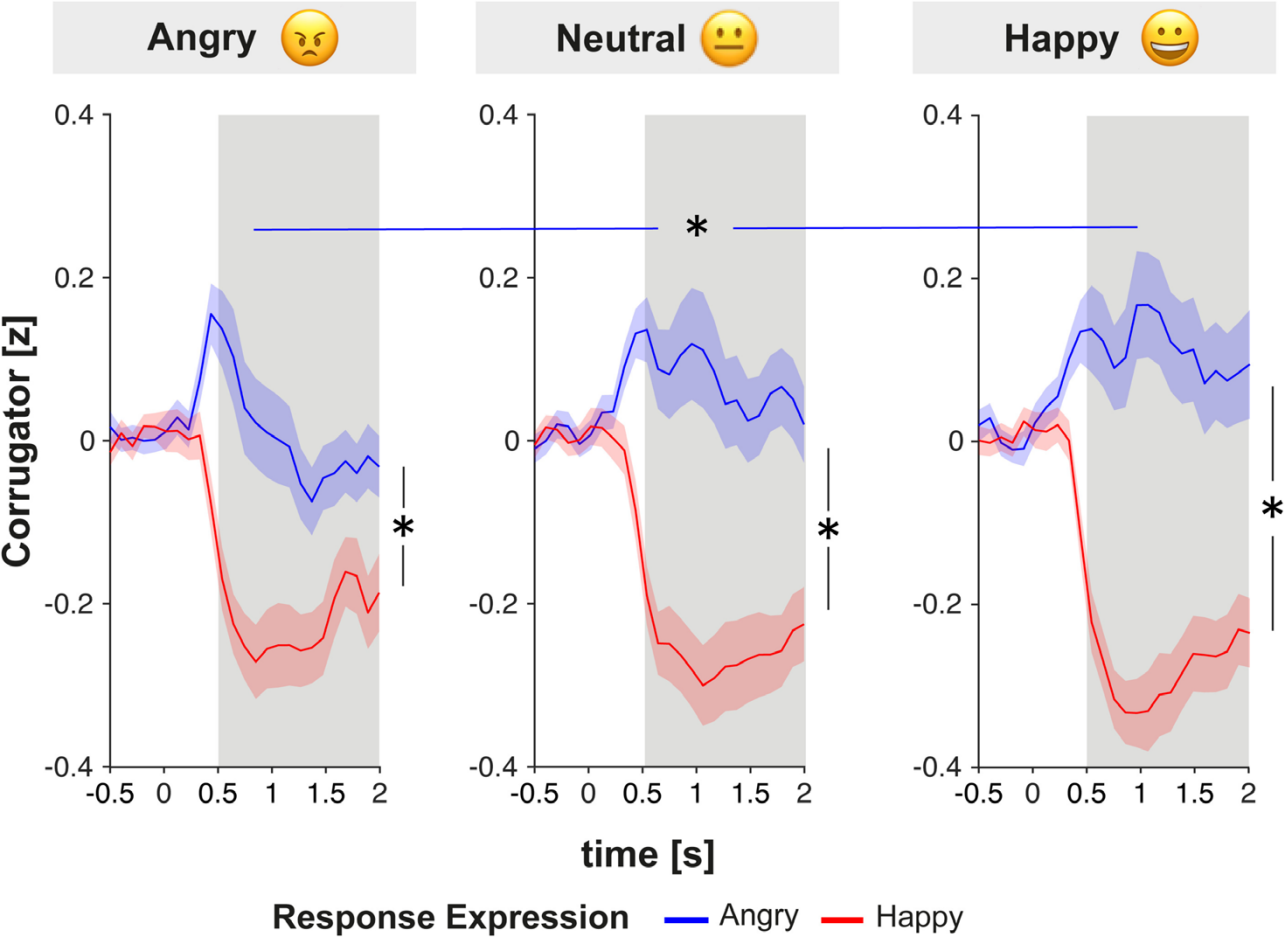
Corrugator activation elicited by the facial emotional expression of the agent as a function of the emoji that was sent by the participant (left: Angry, middle: Neutral, right: Happy). Shaded areas reflect the standard error of the mean.

Analysis of activation in the Corrugator muscle revealed a three‐way interaction between Emoji, Response Expression, and Time, *F*(4.29,293.96) = 3.48, *p* = 0.007, η_p_^2^ = 0.05 (see Figure 5). A step‐down analysis per time window revealed significant interaction effects between *Emoji* and *Response Expression* in all but the first time window (0–500 ms: *F*(2,134) = 0.19, *p* = 0.831, η_p_^2^ < 0.01; 500–1000 ms: *F*(2, 134) = 3.41, *p* = 0.036, η_p_^2^ = 0.05; *F*(2, 134) = 6.99, *p* = 0.001, η_p_^2^ = 0.09; *F*(2, 134) = 4.77, *p* = 0.010, η_p_^2^ = 0.07; see Tables S4 and S5 for full ANOVA results). Hence, post hoc *t*‐tests were conducted in a time window from 500 to 20000 ms post onset of the response expression. Corrugator activity was higher for angry compared to happy response expressions in all emoji conditions (all *p* < 0.001). Additionally, Corrugator activity was significantly higher for angry response expressions when a happy compared to an angry emoji had been sent by the participant, *t*(67) = 2.82, *p* = 0.038, *d* = 0.34. There was no significant difference in Corrugator activity elicited by angry response expressions between happy and neutral emoji conditions, *t*(67) = 1.40, *p* = 0.495, *d* = 0.17, and angry and neutral emoji conditions, *t*(67) = −2.27, *p* = 0.133, *d* = 0.28. There were no other differences between emoji conditions for angry or happy response expressions all (*p* > 0.05). Taken together, Corrugator activity elicited by agents' angry expression was higher when a happy emoji had been sent compared to an angry emoji.

### 3.6 EMG Activity During Sending the Emoji

**FIGURE 6:**
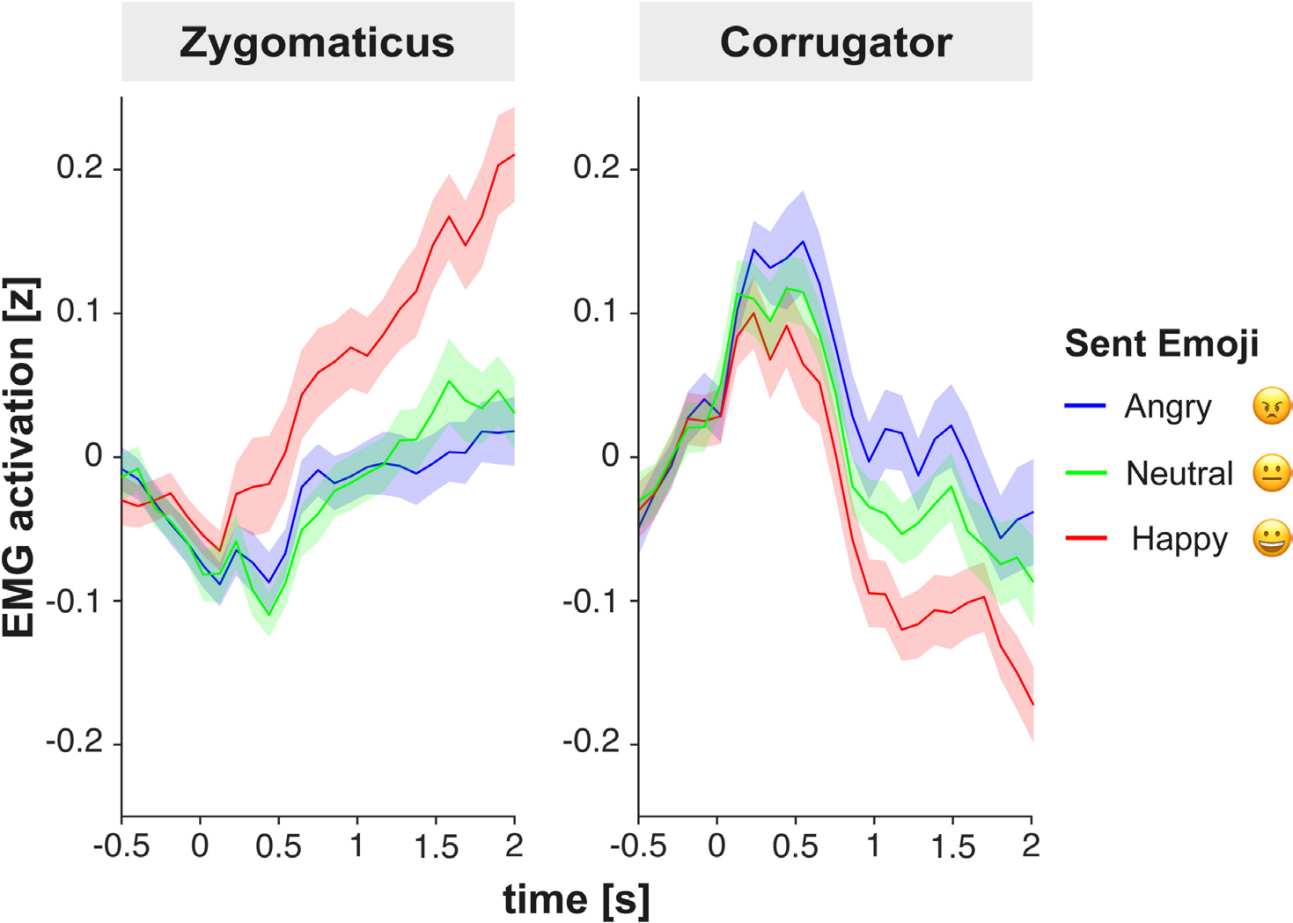
EMG activation of Zygomaticus and Corrugator as a function of the sent emoji. Segments are timelocked to the onset of the cue that instructed participants to send the emoji via button press. Shaded areas reflect the standard error of the mean.

In an exploratory analysis, we also investigated whether sending an emoji via button press would also activate facial muscles (Figure 6). Two additional participants had to be excluded from this analysis because of technical problems with the markers during acquisition.

Zygomaticus activation was analyzed using a repeated measures ANOVA with the factors Emoji and Time (4 levels of non‐overlapping 500 ms windows from 0 to 2000 ms post onset of the cue to send the emoji). The ANOVA showed a significant main effect of Emoji, *F*(1.62, 105.53) = 8.08, *p* = 0.001, η_p_^2^ = 0.11, and a main effect of Time, *F*(1.95, 126.53) = 26.97, *p* < 0.001, η_p_^2^ = 0.29, as well as an interaction between Emoji and Time, *F*(3.44, 223.73) = 4.66, *p* = 0.002, η_p_^2^ = 0.07. A step‐down analysis of the interaction revealed that the effect of *Emoji* was only significant in time windows from 500 to 2000 (all ps < 0.05) but not in the time window from 0 to 500 ms (*p* = 0.103). Post hoc *t*‐tests were therefore conducted for data averaged between 500 and 2000 ms. Sending a happy emoji resulted in higher Zygomaticus activation compared to sending an angry emoji, *t*(65) = 3.58, *p* = 0.002, *d* = 0.44, and sending a neutral emoji, *t*(65) = 3.10, *p* = 0.006, *d* = 0.38, but there was no significant difference between sent‐angry and sent‐neutral emojis, *t*(65) = −0.21, *p* = 0.838, *d* = 0.03.

The repeated measures ANOVA conducted for the Corrugator muscle revealed a main effect of Emoji, *F*(1.64, 106.85) = 4.71, *p* = 0.016, η_p_^2^ = 0.07, and Time, *F*(1.89, 122.59) = 28.99, *p* < 0.001, η_p_^2^ = 0.31, but no interaction effect, *F*(4.31, 280.37) = 1.73, *p* = 0.138, η_p_^2^ = 0.03. Post hoc t‐tests showed higher Corrugator activation for sending an angry compared to sending a happy emoji, *t*(65) = 2.53, *p* = 0.041, *d* = 0.31, but no difference between sent‐angry and sent‐neutral emojis, *t*(65) = 1.53, *p* = 0.131, *d* = 0.19, and sent‐neutral and sent‐happy emojis, *t*(65) = 1.98, *p* = 0.103, *d* = 0.24.

Overall, sending a smiley emoji activated the Zygomaticus muscle, while sending a frowny emoji activated the Corrugator muscle.

## 4 Discussion

The present study investigated whether communicating an emotional intent in face‐to‐face social interactions influenced processing of an interactive partner's response when the communicated intention was signaled by sending an emoji. The study conceptually replicates previous findings where emotional intentions were communicated not via emojis but via actual facial emotional expressions (Kroczek and Mühlberger 2022, 2025). Signaling an affiliative intent via a smile emoji compared to signaling a non‐affiliative intent via an angry emoji thus increased pleasantness and mimicry of affiliative response expressions. These findings demonstrate that communicating an intention is sufficient to modulate the evaluation of an emotional response expression during interaction and do not require the overt display of a facial expression. We also observed a modulatory effect on the evaluation of non‐affiliative response expressions; that is, angry responses were experienced as more pleasant when participants had first sent an angry compared to a smile emoji. Furthermore, increased corrugator activation was elicited by agents display of an angry expression when the expression followed the sending of a smile emoji compared to the sending of an angry emoji. Overall, these data support the view that the evaluation of interactive facial expressions is strongly governed by the context of one's own communicated emotional intentions.

An important question of the present study is whether being instructed to send a particular emoji reflects an emotional intention. Following an instruction to send an emoji is unlikely to evoke a full emotional intention. Yet, the display of an emotional expression and the emotional state of the expresser often diverge (Côté 2005). Here, the crucial aspect is that the expressions are displayed (Van Kleef and Côté 2022) and that the expressed emotions are linked to specific social motives (Parkinson 2005). This means that even when participants did not actually intend to affiliate, they processed the response with respect to the affiliative signal they displayed. This highlights the social‐communicative function and inferential processes elicited by interpersonal exchanges (Van Kleef and Côté 2022). Similarly, even when participants did not believe that the virtual agents actually had an affiliative intention when displaying a smile, they still observed, evaluated, and physiologically responded to the communicative display of an emotion. Nevertheless, testing the observed effects in experimental tasks in which genuine affiliative intentions and social goals are manipulated will be important to evaluate social‐functional explanations of interactive social behavior.

Mimicry responses have often been discussed as affiliative signals which strengthen social connections and influence interpersonal relations (Chartrand and Lakin 2013). It has also been argued that mimicry may only occur when a person has an affiliative intent (Hess 2021). These claims were also supported in interactive paradigms where sending an affiliative (smile) compared to non‐affiliative (frown) expression increased the pleasantness of the interaction as well as Zygomaticus activation elicited by the response expression of an interactive partner (Kroczek and Mühlberger 2022, 2025). The present study conceptually replicates these findings using a different social cue and thus demonstrates that the affiliative effect may be mostly driven by the communicative intention rather than the overt display of a facial expression. However, it should be noted that two findings of the current study do not directly support this claim. First, participants also rated interactions as more pleasant when the agents responded with an angry expression after participants had sent an angry compared to a happy emoji. This effect was already observed in the study by Kroczek and Mühlberger (2022) and might be related to reward processing that is elicited when one's own communicative signal is reciprocated by another person (Pfeiffer et al. 2014). Engaging in reciprocal social interaction might therefore be rewarding even when the valence of the interaction is negative. It should be noted, however, that the effect sizes of this anger congruency effect were smaller than the happy congruency effect. Another finding that challenges the affiliative mechanisms of communicative intentions is that we observed higher Corrugator activation elicited by the agent's angry response expression when participants had first sent a happy compared to an angry emoji. Complementary to the anger congruency effect in the valence rating, this effect might indicate an aversion towards incongruent exchanges in social interactions. This might also be related to violations of social conventions; that is, having one's smile responded to with an angry expression might be perceived as unpredicted and rude (Turner 2002). This alternative explanation aligns with the EASI model in a sense that incongruent emotional expression could elicit inferential processing about the other person. The fact that this effect was not observed in the previous studies might be explained by decreased sensitivity in EMG measures when participants actually displayed facial expressions. Taken together, the influence of sending an emotional intention on the evaluation of the response might underlie an affiliative mechanism but also reflect evaluation of the fit between sending and receiving.

Adding to previous findings (Kroczek and Mühlberger 2022, 2025) the current study demonstrates that communicating emotional intentions can also affect processing of response expressions when they are signaled via digital; that is, non‐bodily channels. This has implications for our understanding of the mechanism by which communicative intentions influence social processes. Notably, even though participants were not instructed to display facial expressions, we found that sending an emoji via button press did also activate corresponding facial muscles; that is, when participants sent a smile emoji, there was increased activation in the Zygomaticus muscle, and when participants sent a frown emoji, there was increased activation in the Corrugator muscle. This represents a novel physiological effect of digital communication that may underlie the role of emojis as emotional expressions (Erle et al. 2022). This makes it likely that the observed interaction between sending and receiving emotional signals may be driven (at least partly) by proprioceptive feedback signals from facial muscles. Although the facial feedback hypothesis has been controversial (Coles et al. 2019, 2022; Wagenmakers et al. 2016) recent evidence from a study using facial neuromuscular stimulation suggests that muscular activation can influence emotional experience (Efthimiou et al. 2024) and is thus a possible mechanism for the emotional effects in social interactions. However, it is important to note that our results cannot rule out alternative mechanisms where social intentions may have an impact via the activation of higher‐level representations (Kroczek et al. 2021; Tucciarelli et al. 2019; Wurm et al. 2017). For instance, the selection of an emoji might have activated an emotional higher‐order representation, which then modulates activity in facial muscles as a downstream process. In this explanation, the activation of facial muscles would not be required for the interaction between sending and receiving but rather would be a by‐product of the activation of a higher‐level concept. Future studies might experimentally stimulate neuromuscular activity to test causal effects of facial feedback and communicative intentions in social interactions. In summary, the present results show that even when no overt facial expressions are displayed, sending an emotional signal still activates corresponding facial expressions, suggesting a physiological mechanism of interactive emotions. Interestingly, this mechanism may also underlie digital communication.

To our knowledge, the current study is the first to measure physiological mechanisms of social interaction using digital communication. However, there are some limitations that need to be discussed. First, the use of emojis, as implemented in the present study, does not represent how emojis are used in everyday communication. Emojis are typically not used in face‐to‐face interactions but rather in text‐based interaction (Cherbonnier and Michinov 2022)—although they are used in face‐to‐face video conferences. Nevertheless, emojis are communicative signals and express emotional intentions (Erle et al. 2022). Ratings in the present study also confirmed that three emojis were associated with different levels of valence. Consequently, while the experimental setting is artificial in this regard, emojis can still be seen as valid communicative signals.

Another limitation relates to the setting of the screen‐based face‐to‐face interaction. To increase experimental control, participants were instructed which particular emoji they had to send during the interaction with the agent. Sending an instructed emoji does therefore not necessarily reflect the participant's emotional intention. Notably, as the emoji was sent in a social interaction, it still reflects a communicative intention. Even though participants might not actually have had a particular intention towards the agent, they were still aware that sending an emotional expression towards another person would make the other person infer a particular intention in the sender (Lehmann et al. 2023; Van Kleef 2009). To further investigate the effect of intentionality, future studies should incorporate conditions in which participants are free to choose which or whether at all they want to send an emotional signal towards an interactive partner.

In summary, substituting facial emotional expressions with emojis in a face‐to‐face social interaction allowed us to investigate the mechanisms by which sending an emotional signal influences evaluation and mimicry of an emotional response. Sending an emoji resulted in activation of the corresponding facial muscles. Furthermore, sending an affiliative signal increased the experienced pleasantness and activation in the Zygomaticus muscle for happy facial response expressions. This suggests that senders might use proprioceptive feedback to influence the processing of subsequent social–emotional signals. In addition, our findings show that senders were sensitive to the congruency between the sent and the received signal, indicating that interactive exchanges are constantly evaluated for appropriateness.

## Author Contributions

**Leon O. H. Kroczek:** conceptualization, methodology, data curation, formal analysis, supervision, visualization, project administration, writing – original draft. **Silke Frank:** data curation, investigation. **Uta Gold:** data curation, investigation. **Fridolin Hesse:** data curation, investigation. **Selina Hettenkofer:** data curation, investigation. **Nadja Peterreins:** data curation, investigation. **Lorenz Teutsch:** data curation, investigation. **Valerie Theophile:** data curation, investigation. **Andreas Mühlberger:** writing – review and editing.

## Conflicts of Interest

The authors declare no conflicts of interest.

## Supporting information


**Data S1:** psyp70137‐sup‐0001‐DataS1.pdf.

## Data Availability

The data that support the findings of this study are openly available in OSF at https://osf.io/z8cve/, reference number DOI https://doi.org/10.17605/OSF.IO/Z8CVE.
