# Supplementary material for: Communicative Social Intentions Modulate Emotional Mimicry Responses
Source: Psychophysiology. 2025 Sep 9;62(9):e70137. doi: 10.1111/psyp.70137 (PMC12421215; doi:10.1111/psyp.70137)
Supplement: Supplementary file 1 — Data S1: psyp70137‐sup‐0001‐DataS1.pdf. [file PSYP-62-e70137-s001.pdf]

Supplementary Materials: Communicative social intentions modulate emotional mimicry responses

Leon O. H. Kroczek[1*], Silke Frank[1], Uta Gold[1], Fridolin Hesse[1], Selina Hettenkofer[1], Nadja Peterreins[1], Lorenz Teutsch[1], Valerie Theophile[1] & Andreas Mühlberger[1]

[1] Department of Psychology, Clinical Psychology and Psychotherapy, University of Regensburg

Descriptive summary of sample

Table S1. Summary statistics regarding questionnaires. SPIN = Social Phobia Inventory. Positive affect and negative affect are from the PANAS.

Variable	Mean	SD	Min	Max
Age	22.74	5.71	18	50
SPIN	18.39	8.41	2	45
Positive Affect	28.11	5.10	16	40
Negative Affect	13.39	3.25	10	26

Ratings of Agent Facial Expressions after the main experiment

To check whether facial expressions of the virtual agents were experienced as intended an additional rating session was conducted after the main experiment. Here participants were presented with still frames of each agent with angry, neutral, or happy facial expressions and ratings of valence and arousal were obtained (Figure S1).

For valence ratings there was a significant main effect of *Agent*, $F(2.41, 161.24) = 8.26$, $p < .001$, $\eta_p^2 = .11$, a significant main effect of *Emotion*, $F(1.34, 89.46) = 180.36$, $p < .001$, $\eta_p^2 = .73$, and a significant interaction between *Agent* and *Emotion*, $F(5.17, 346.55) = 7.09$, $p < .001$, $\eta_p^2 = .10$. Importantly, for each agent the happy expression was experienced as more pleasant than the neutral expression (all $p < .001$), while the angry expression was experienced as more unpleasant than the neutral expression (all $p <.001$). Post-hoc analysis revealed that the interaction was driven by more positive valence ratings for happy and neutral expressions of the F1 agent compared to the other agents (all $p < .05$).

With respect to arousal ratings we found a main effect of *Emotion*, $F(2, 134) = 30.42$, $p < .001$, $\eta_p^2= .31$, but no main effect of *Agent*, $F(3, 201) = 0.76$, $p = .520$, $\eta_p^2 = .01$, and no interaction, $F(4.19, 281.00) = 2.05$, $p = .084$, $\eta_p^2= .03$. Post-hoc tests revealed that both angry and happy expressions were experienced as more arousing than neutral expression (all $p < .001$), and that angry expressions were experienced as more arousing than happy expressions ($p = .026$).

Overall, the post-experimental ratings demonstrate that the facial emotional expressions were experienced as intended.

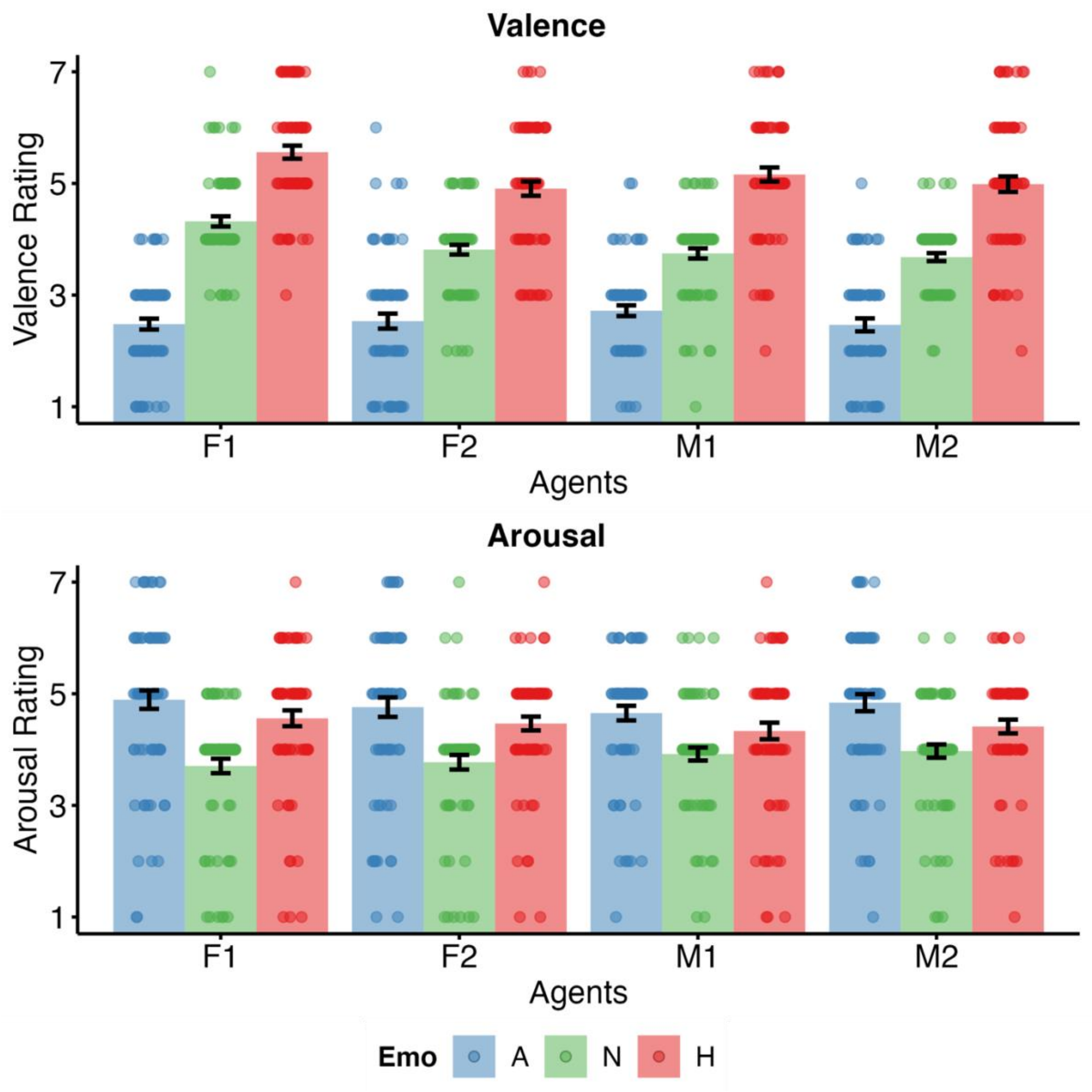

Figure S1: *Valence and Arousal ratings of each agent with angry, neutral, or happy facial expressions in the post-experimental rating. Ratings were obtained with respect to still frames showing agents with specific expressions. Error bars reflect the standard error of the mean.*

ANOVA tables: EMG effect of Time, Emoji, Response Expression

Zygomaticus

Table S2: Zygomaticus Muscle ANOVA Time x Emoji x Response Expression – timelocked to Agent Expression

**Predictor**	df_{Num}	df_{Den}	F	p	η^2_p
Response Expression	1	67	19.82	<.001	.23
Emoji	2	134	0.60	.547	<.01
Time	2.04	136.94	31.47	<.001	.32
Emoji x Response Expression	2	134	6.10	.003	.08
Emoji x Time	4.15	277.89	1.29	.271	.02
Response Expression x Time	1.88	125.78	16.96	<.001	.20
Emoji x Response Expression x Time	4.16	278.41	4.14	.002	.06

Table S3: Zygomaticus Muscle ANOVAs Emoji x Response Expression for each 500 ms time window – timelocked to Agent Expression

**Predictor**	df_{Num}	df_{Den}	F	p	η^2_p
		**0 – 500 ms**			
Emoji	1.82	121.93	0.19	.810	<.01
Response Expression	1	67	0.21	.648	<.01
Emoji x Response Expression	2	134	0.03	.975	<.01
		**500 – 1000 ms**			
Emoji	2	134	2.02	.136	.03
Response Expression	1	67	14.93	<.001	.18
Emoji x Response Expression	2	134	4.39	.014	.06
		**1000 – 1500 ms**			
Emoji	2	134	0.93	.398	.01
Response Expression	1	67	22.51	<.001	.25
Emoji x Response Expression	2	134	8.93	<.001	.12
		**1500 – 2000 ms**			
Emoji	2	134	0.28	.753	<.01
Response Expression	1	67	18.61	<.001	.22
Emoji x Response Expression	2	134	4.52	.013	.06

Corrugator

Table S4: Corrugator Muscle ANOVA Time x Emoji x Response Expression – timelocked to Agent Expression

**Predictor**	df_{Num}	df_{Den}	F	p	η^2_p
Response Expression	1	67	35.72	<.001	.35
Emoji	1.75	117.13	0.65	.504	<.01
Time	1.97	131.80	14.54	<.001	.18
Emoji x Response Expression	2	134	5.52	.005	.08
Emoji x Time	3.90	260.99	1.28	.279	.02
Response Expression x Time	1.78	119.15	15.96	<.001	.19
Emoji x Response Expression x Time	4.39	293.96	3.48	.007	.05

Table S5: Corrugator Muscle ANOVAs Emoji x Response Expression for each 500 ms time window – timelocked to Agent Expression

**Predictor**	df_{Num}	df_{Den}	F	p	η^2_p
		**0 – 500 ms**			
Emoji	2	134	0.04	.962	<.01
Response Expression	1	67	25.18	<.001	.27
Emoji x Response Expression	2	134	0.19	.831	<.01
		**500 – 1000 ms**			
Emoji	1.84	123.18	0.38	.665	<.01
Response Expression	1	67	41.65	<.001	.38
Emoji x Response Expression	2	134	3.41	.036	.05
		**1000 – 1500 ms**			
Emoji	2	134	2.44	.091	.04
Response Expression	1	67	26.95	<.001	.29
Emoji x Response Expression	2	134	6.99	.001	.09
		**1500 – 2000 ms**			
Emoji	1.74	116.82	0.28	.727	<.01
Response Expression	1	67	22.31	<.001	.25
Emoji x Response Expression	2	134	4.77	.010	.07
